# Photosensitive Thin Films Based on Drop Cast and Langmuir-Blodgett Hydrophilic and Hydrophobic CdS Nanoparticles

**DOI:** 10.3390/nano10122437

**Published:** 2020-12-05

**Authors:** Momoka Nagamine, Magdalena Osial, Justyna Widera-Kalinowska, Krystyna Jackowska, Paweł Krysiński

**Affiliations:** 1 Department of Chemistry, Adelphi University, One South Avenue, Garden, NY 11530, USA; mjn5615@psu.edu (M.N.); widera@adelphi.edu (J.W.-K.); 2 Faculty of Chemistry, University of Warsaw, Pasteura 1, 02-093 Warsaw, Poland; kryjacko@chem.uw.edu.pl (K.J.); pakrys@chem.uw.edu.pl (P.K.)

**Keywords:** CdS, thin films, photoelectrochemical studies, langmuir-blodgett technique

## Abstract

Comparative photoelectrochemical studies of cadmium sulfide (CdS) nanoparticles with either hydrophilic or hydrophobic surface properties are presented. Oleylamine organic shells provided CdS nanoparticles with hydrophobic behavior, affecting the photoelectrochemical properties of such nanostructured semiconductor. Hydrophilic CdS nanoparticles were drop-cast on the electrode, whereas the hydrophobic ones were transferred in a controlled manner with Langmuir-Blodgett technique. The substantial hindrance of photopotential and photocurrent was observed for L-B CdS films as compared to the hydrophilic, uncoated nanoparticles that were drop-cast directly on the electrode surface. The electron lifetime in both hydrophilic and hydrophobic nanocrystalline CdS was determined, revealing longer carrier lifetime for oleylamine coated CdS nanoparticles, ascribed to the trapping of charge at the interface of the organic shell/CdS nanoparticle and to the dominant influence of the resistance of the organic shell against the flux of charges. The “on” transients of the photocurrent responses, observed only for the oleylamine-coated nanoparticles, were resolved, yielding the potential-dependent rate constants of the redox processes occurring at the interface.

## 1. Introduction

In the 21st century, humanity is facing two major challenges: energy crisis and environmental problems. Due to the significant increase of carbon dioxide emissions into the atmosphere, which results in escalation of the greenhouse effect, the development of an alternative source of energy is a very important research topic aimed at alleviating the above mentioned challenges. Among all types of alternative sources of energy, solar energy is the most abundant and efficient one, so the development of nanostructured photoactive materials capable of efficiently utilizing solar energy for clean fuel production has been recently a major focus of the scientific community.

Semiconductor nanoparticles or quantum dots (QDs) are known to be effective materials for photovoltaic [1,2,3,4,5,6] applications due to their unique properties, such as high extinction coefficient of light absorption, size and shape-controlled tunable bandgap energy and carrier multiplication effect and so forth. Various configurations of QD based photovoltaic cells [7] have been studied. Those include QDSC—quantum dots sensitized solar cells—where QDs act as a light harvester [8,9,10,11] and QD-layer based solar cells, where QDs colloidal layer is responsible for both light-harvesting and charge transport [12,13,14]. There are many reports in the literature describing functionalization of nanoparticles with various organic species, including thiols [11,12,15,16], amines [17] and carboxylic acids [18] that passivate the surface states of QDs [13], introduce electrical dipoles [15] and act as electron traps [14] or hole traps [16]. Such functionalization by organic coating also provides means of coupling and organizing nanostructured semiconductors onto the electrodes in the semiconductor-sensitized solar cells (SSC), charge transfer-based sensors and optoelectronic devices [16]. In this context, cadmium chalcogenides semiconductors (CdS, CdSe and CdTe) are widely studied due to their low production cost and facile synthesis process [19,20]. CdS is one of the important II-VI semiconducting materials with 2.42 eV direct bandgap corresponding to the visible part of the electromagnetic spectrum [21]. Due to this fact, it is widely used in photovoltaics [22,23] and photocatalysis [24,25]. The production of semiconductor QDs or thin layers is significantly cheaper compared to their bulk counterparts since their synthesis takes place at significantly lower temperatures and with solution-based approaches. CdS QDs can be easily obtained with various methods [26,27,28,29,30,31,32,33,34,35,36] using single source precursor [26,30], cyclic microwave radiation [27], chemical vapor deposition route [28,29], sonochemical [31,33] or reverse micelle method [32], solvothermal [34] or hydrothermal [35] methods as pure CdS NP, using surface modifiers [36] or in the form of core/shell NP [37]. The growth of protective layer on the nanoparticles has demonstrated to be an effective solution to eliminate the surface defects by reducing the number of dangling bonds on surface and improve the photo stability by physically separating the core surface from its surrounding medium [38,39]. Optimization of three aspects of the NP toward an increase in cell performance focuses on: (i) materials, including not only light-absorbing material but also electron and hole conductors, (ii) control of recombination and band alignment by surface treatments and (iii) development of absorbing nanocomposites with enhanced light-harvesting and collecting properties.

The processes that occur at the semiconductor nanoparticle interface, such as charge carrier recombination and charge transfer across the interface, are strongly affected by the interface structure and presence of organics [40]. Those interactions are complex, not yet fully understood. Still, they have fundamental implications in the development of SSCs. Therefore, the rationale of this paper was to compare the properties of uncoated, hydrophilic CdS nanoparticles drop-cast on the electrode surface, with hydrophobic, oleylamine-coated CdS particles, organized in layers on the electrode surface with the Langmuir-Blodgett method. By using simple measurements of the open circuit (OCP), photocurrent decays under illumination and literature models, we tried to extract information about the effect of organic layer modification on the electron lifetime and the recombination and charge transfer processes occurring in these two systems. We used long aliphatic chain oleylamine molecules as the surface modifier, because this molecule easily forms the well-ordered Langmuir films that can be subsequently transferred in a controlled manner onto the conducting substrate via the Langmuir-Blodgett technique. The Langmuir-Blodgett technique [41] allows for the deposition of ultra-thin, highly ordered films. It also permits the tuning of the light-harvesting behavior with the increase in the number of nanoparticle layers. Therefore, we used this technique in an attempt to analyze the effect of organic coating on the performance of such prepared photoanodes and to provide the principal knowledge regarding such effect on electron transfer process. The substantial decrease of photoactivity for the case of L-B CdS films was observed. This effect was most probably due to the resistance of dense organic film of oleylamine, inhibiting the charge transfer of minority charges (holes) to the redox solution and slowing down the electron-hole recombination process, most probably by creating more charge carrier traps within the hydrophobic nanoparticles. Better understanding of the excited-state interfacial electron-transfer reactions at the interface of semiconductor nanoparticle-shell molecule-conducting substrate are also of interest from a fundamental standpoint. This paper gives an insight into these important basic studies.

## 2. Experimental Results

### 2.1. Chemicals

Cadmium acetate (Cd(CH_3_COO)_2_), sodium sulfide nonahydrate (Na_2_S∙9H_2_O), sodium sulfate (Na_2_SO_4_) and sodium sulfite (Na_2_SO_3_), ethanol, oleylamine (OA) and hexane were all purchased from Sigma-Aldrich. All the chemicals were used without further purification. All solutions were prepared using Milli-Q water.

### 2.2. Synthesis of CdS Hydrophilic Nanoparticles and Hydrophobic NPs Formation

To synthesize CdS nanoparticles, 100 mL of 0.01 M Cd(CH_3_COO)_2_ precursor [42] in 1:1 water-ethanol solution was heated at 80 °C, then 100 mL of 0.01 M Na_2_S in 1:1 water-ethanol solution was slowly added to the cadmium precursor solution, with continuous stirring at 1000 rpm, until the stoichiometric amount was finally achieved. As a result, the yellow precipitate started to form. The suspension was heated for 2 h at 80 °C with stirring based on the procedure described in literature [43]. The obtained precipitate was washed with water and ethanol four times to remove impurities. Finally, the suspension was centrifuged for 30 min at 13,000 rpm and the settled nanoparticles were collected and dried overnight in a hot air oven at 50 °C for 12 h. The synthesized hydrophilic nanoparticles were transferred to hydrophobic phase as follows: 1 mg of the as-synthesized CdS nanoparticles were suspended in 2 mL of deionized water and sonicated for 15 min, yielding relatively stable colloidal suspension. 2 mL of hexane was added to the solution, followed by 50 μL of oleylamine. The biphasic system was vigorously shaken for 5 min, resulting in the transfer of majority of the nanoparticles into the top, organic phase. The top layer was washed with ethanol and used for the deposition of the L-B film on a cleaned ITO (indium tin oxide coated glass). ITO substrates were cleaned by immersing in a mixture of 2:1 H_2_O_2_ and NH_4_OH and sonicated for 5–10 min.

### 2.3. Langmuir Film Formation and Langmuir-Blodgett (L-B) Deposition of Hydrophobic CdS Nanoparticles

The Langmuir-Blodgett trough by NIMA Technology was used to deposit CdS monolayers on ITO substrates. The samples were prepared with a specified number of layers by subsequent dip-withdraw Langmuir-Blodgett cycles controlled by NIMA software at 25 mN/m surface pressure of the pre-formed Langmuir films of OA-coated CdS nanoparticles. Long aliphatic chain oleylamine molecules were used as the surface modifier to form the well-ordered Langmuir films that were subsequently transferred in a controlled manner onto the conducting substrate via the Langmuir-Blodgett technique. The Langmuir-Blodgett technique allowed for the deposition of ultra-thin, highly ordered films with the increasing number of nanoparticle layers.

#### 2.3.1. Langmuir Film Formation

**Figure 1:**
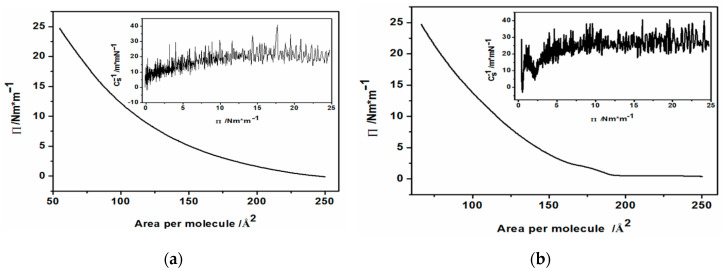
The isotherms of (**a**) oleylamine and (**b**) CdS nanoparticles capped with oleylamine. The insets show compressibility moduli vs. Π for pristine oleylamine and CdS.

The Langmuir films of CdS hydrophobic, oleylamine-coated nanoparticles were prepared following the well-known procedures of Langmuir film formation using amphiphilic molecules [41,44]. Briefly, after cleaning the trough with chloroform, ethanol and finally, with Milli Q water, the Langmuir trough was filled with ca. 250 mL of triple-distilled water to form a positive meniscus. Then, the surface pressure sensor equipped with a Wilhelmy plate was zeroed and a specified amount of hexane suspension of hydrophobic nanoparticles (1.67 mg/mL) was applied onto the surface of the water. The system was left to evaporate hexane solvent and equilibrate. The solvent evaporated within a short time (ca. 10 min) leaving the nanoparticles spread over the whole water surface. For all the Langmuir and Langmuir-Blodgett experiments here, 300 µL of suspension was used and added separately in 6 batches in 10 min intervals. The nanoparticles were compressed by moving the barriers of the trough inwards at ambient temperature, with continuous monitoring of the surface pressure, until the desired value of 25 mN/m was reached. This was done in hope to align the hydrophobic nanoparticles in a regular arrangement. During the whole compression cycle the changes of surface pressure were plotted against the decreasing area of the trough, resulting in a so-called pressure-area isotherm. For comparison, Langmuir isotherm for pure oleylamine was also recorded. In this case, 15 µL of oleylamine in hexane solution (2 mg/mL) was applied onto the air/water interface in the trough. The resultant so-called Langmuir isotherms for pure oleylamine and oleylamine-capped semiconductor nanoparticles are exemplified in Figure 1a,b.

The recorded pressure-area isotherms contain information on the organization and interactions of hydrophobic, oleylamine-coated nanoparticles at various stages of compression and distance between the nanoparticles (or oleylamine molecules in the case of pure oleylamine).

As can be seen in this figure, the isotherm of pristine oleylamine (Figure 1a) shows a typical behavior of Liquid Expanded (LE) monomolecular films with no visible transition to a more organized structures under the ambient temperature. The compressibility modulus defined as:

(1) $$C^{-1}\;-\left(\frac{1}{A}\right)\times \left(\frac{dA}{d\pi }\right)$$

of the oleylamine monolayer reached the value of ca. 25 m/mN (Figure 1a inset). This value is characteristic for LE molecular films. The Langmuir isotherm in the case of oleylamine-capped CdS nanoparticles shows a different behavior. Below the surface pressure of 5 mN/m, corresponding to the area 190–160 cm^2^ available for the nanoparticles on the surface of the trough limited by the moving barriers of the trough, one can observe a small plateau. This is followed by a minimum of the compressibility modulus (Figure 1b, inset). With further compression by the barriers, the isotherm rises to the pre-determined surface pressure, showing only slightly higher compressibility modulus (ca. 30 m/mN) than that of pristine oleylamine. Typically, the appearance of the observed plateau indicates a phase transition and a coexistence of the LE and liquid condensed (LC) phases. However, we think that this is due to the aggregation of hydrophobic nanoparticles. This may also be facilitated by the hydrophobic interactions of the oleylamine molecules from the hydrophobic shell of the CdS core. Furthermore, we believe that there is a remaining substantial amount of unwashed, free OA molecules applied simultaneously on the interface, together with CdS nanoparticles. We could not avoid such a situation, because extensive washing of the hydrophobic nanoparticles with an abundance of ethanol could destabilize their suspension in hexane since OA was only physically adsorbed on the CdS surface. And indeed, close inspection of the surface of the subphase in the trough revealed aggregating structures being formed during the compression. These structures did not merge into one smooth surface at the final pressure of 25 mN/m. Nevertheless, we tried to use the Langmuir-Blodgett technique to transfer these surface structures onto the ITO substrate.

#### 2.3.2. Langmuir-Blodgett Film Transfer

The Langmuir-Blodgett (L-B) transfer was carried out under the pressure control mode of the trough software. The L-B transfer was done with “downstroke” mode first, that is, by dipping the clean ITO substrate through the compressed layer of hydrophobic nanoparticles (first layer) and then “upstroke” for the second layer. Thus, all even numbers of transfers correspond to the sample being withdrawn from the trough through the compressed layer of nanoparticles. All strokes were carried out while keeping the 25 mN/m surface pressure, as mentioned above. So, as the dipper is moving down and up regularly, the barriers move in linearly to compensate for the removal of the monolayer from the surface of the subphase onto the ITO substrate. Repeated downstrokes and upstrokes of the substrate resulted in the deposition of multilayer structures. After the final upstroke, the sample was left to dry under ambient atmosphere for 24 h.

The quality of the deposits was first examined by comparing the area of the ITO that should be covered with a layer with the area of the trough that was decreased by the barriers moving inward to maintain the constant surface pressure. If both areas are equal, this will mean that the monolayer was transferred onto the substrate, covering 100% of its surface, yielding the so-called transfer ratio (TR) equal to 1. Unfortunately, while the first two transfers (first the downstroke and then the upstroke) resulted in almost complete coverage of the substrate, with TR value close to unity, the subsequent strokes yielded continuously diminishing transfers from the surface of the trough onto the ITO. We think that this may imply patchy film adhesion and/or aggregation of the deposits on the ITO surface.

### 2.4. Methods of Characterization

Malvern Instruments Zetasizer Nano ZS apparatus was used to evaluate the hydrodynamic size (Dynamic Light Scattering (DLS) measurement) and zeta potential of the nanoparticles. The samples were prepared by suspending the as-synthesized nanoparticles in 6 mL of deionized water and sonicating for 15 min.

The crystallinity of nanoparticles was confirmed by obtaining the powder X-ray diffraction (PXRD) patterns collected on a Bruker D8 Discover diffractometer with Debye-Scherrer geometry at room temperature. Measurements were performed using Cu Kα radiation (λ = 1.540598 Å) at a scan rate of 1°/min in 0.012° steps, covering a range of 2Θ from 20° to 130°.

The morphology of the synthesized nanoparticles was examined using a Scanning Electron Microscope (SEM), (Merlin, Zeiss, Stuttgart, Germany) equipped with Energy Dispersive X-Ray Spectroscopy (EDS) head.

Fourier transform infrared spectroscopy (FTIR) was carried out using NicoLet iN 10 Infrared Microscope by ThermoFisher Scientific to confirm the formation of an organic shell.

Transmission Electron Microscopy (TEM), (Libra 120 Plus, Zeiss, Stuttgart, Germany) investigations were conducted on FEI Talos F200X Transmission Microscopes at 200 KV. The measurements were performed in the TEM and STEM mode using High Angle Annular Dark Field (HAADF) detector.

Chemical composition of the samples was determined using Energy Dispersive X-ray Spectroscopy (EDS) using Brucker BD4 instrument. The samples for TEM were prepared by dropping the colloid dispersion on a carbon film supported on a 300-mesh copper grid. X-ray photoelectron spectroscopy (XPS) spectra were acquired using a Kratos Axis Supra spectrometer (Kratos Analytical Ltd., Manchester, UK) equipped with a monochromatic Al Kα (1486.7 eV) radiation source. The instrument work function was calibrated to give a binding energy (BE) of 84.0 ± 0.1 eV for the 4f_7/2_ line of metallic gold and the spectrometer dispersion was adjusted to give a BE of 932.62 eV for the Cu 2p_3/2_ line of metallic copper. Energy resolution was examined on silver sample. Survey (wide) spectra were collected with a quality corresponding to the full width at half maximum (FWHM) parameter for Ag 3d line equal to 0.71 eV at energy step size equal to 0.5 eV. For high resolution spectra the FWHM parameter for Ag 3d line was equal to 0.58 eV at step size was: 0.1 eV. Space Kratos patented charge neutralizer system was used to reduce charge compensation. The peak fitting and deconvolution were conducted using CasaXPS software version 2.3.18 and a Shirley background based on Gaussian-Lorentzian (30) function was applied. All spectra were calibrated using the adventitious C 1s peak with a fixed value of 284.8 eV.

Atomic Force Microscopy (AFM) microscope was used for imaging and thickness measurements by AFM scratching technique. The imaging, as well as scratching, was done using FMV-A silicon cantilevers (Bruker Corporation, Billerica, MA, USA) with a spring constant, k ∼ 4.8 Nm^−1^. Morphology of the studied area was imaged using PeakForce Tapping^®^ Mode (Bruker Corporation, Billerica, MA, USA). Subsequently, part of a sample was removed in Contact-Mode from a smaller square-shaped area, chosen for thickness measurement. It was done by applying a much higher deflection setpoint voltage to increase contact force that occurs between an AFM tip and a sample. Morphology of the previously imaged large area was again monitored in PeakForce Tapping^®^ Mode. The morphology changes let to estimate the thickness value of the deposited material.

UV-vis absorption spectra of the synthesized nanoparticles were obtained using a Perkin Elmer Lambda 35 to estimate the bandgaps of the nanoparticles. Photoluminescence studies were carried out using a Fluorolog-3 spectrofluorimeter (Jobin Yvon Inc., Newark, NJ, USA), operating at 405 nm excitation wavelength. Capacitance recordings for the Mott-Schottky analysis were performed at 100 Hz with AC voltammetry technique (CH Instruments 660C) with a typical three-electrode system.

### 2.5. Photoelectrochemical Measurements

For photoelectrochemical studies, a three-electrode cell was used with a quartz window. CdS hydrophilic drop-cast nanoparticles or CdS LB films formed from hydrophobic nanoparticles coated ITO glass served as the working electrode. Pt wire and mercury/mercurous sulfate electrode (MSE, sat. K_2_SO_4aq_, +0.64 V vs. NHE) were applied as counter and reference electrodes, respectively. A xenon lamp (100 mW/cm^2^) and CHI660D Electrochemical Workstation manufactured by CH Instruments, INC. was used to carry out the photoelectrochemical experiments. Aqueous solution of pH 9.8 containing 0.2 M Na_2_SO_3_ and 0.1 M Na_2_SO_4_ was used as electrolyte.

## 3. Results and Discussion

### 3.1. Physico-Chemical Characteristics of CdS

#### 3.1.1. XRD Analysis

The size and zeta-potential of the as-synthesized hydrophilic CdS nanoparticles were determined by dynamic light scattering (DLS) measurement. The as-synthesized CdS nanoparticles show the zeta potential close to zero, therefore their suspension is unstable and tends to aggregate and precipitate. The size of the hydrophilic nanoparticles was determined to be 5.6 ± 1.7  nm.

**Figure 2:**
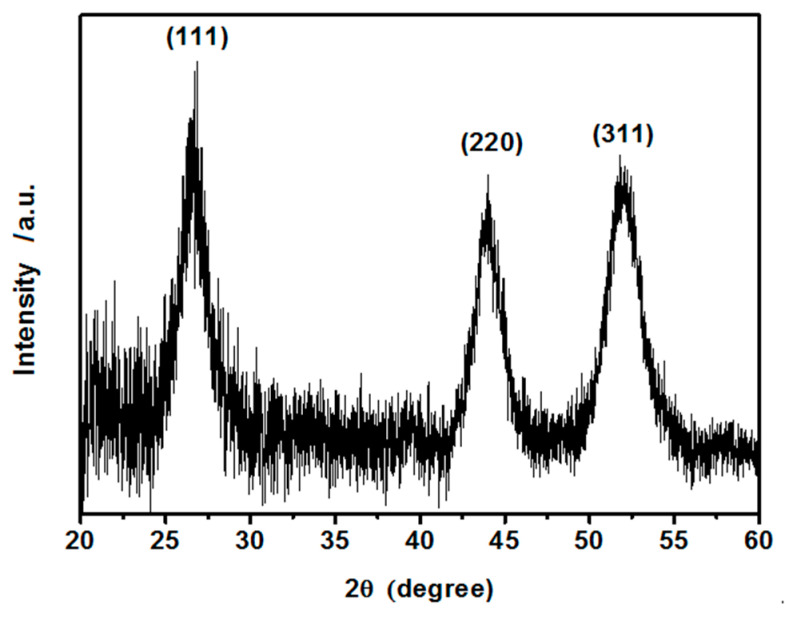
Powder X-ray diffraction pattern of as synthesized CdS crystalline hydrophilic nanoparticles with assigned *hkl* indices.

A representative powder diffraction pattern for the as-synthesized dry hydrophilic CdS nanoparticles is shown in Figure 2. Powder X-ray diffraction (PXRD) pattern confirms good crystallinity with a face-centered cubic crystal structure and was indexed accordingly to the standard International Centre for Diffraction Data (ICDD) card number 10-0454 [45]. No diffraction peaks from other crystallites were detected. A relatively low signal-to-noise ratio suggests that the CdS nanocrystals are small. The average crystallite size was estimated from the Debye-Scherrer formula, yielding ca. 5 nm (±0.7 nm). This result stands in good agreement with the size determined by the DSL method.

#### 3.1.2. XPS Analysis

**Figure 3:**
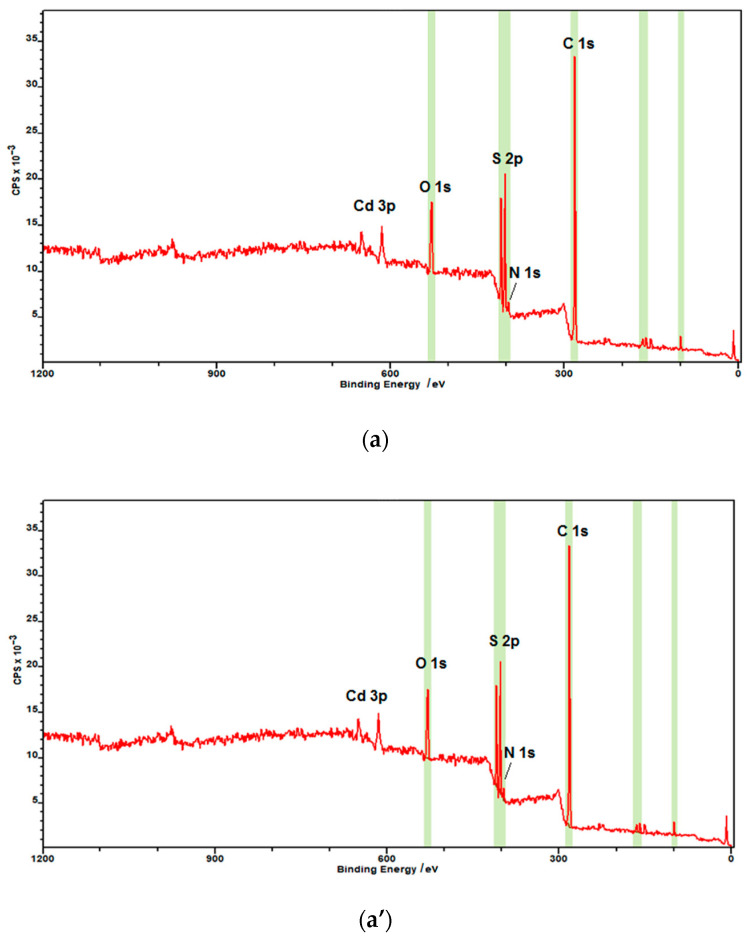

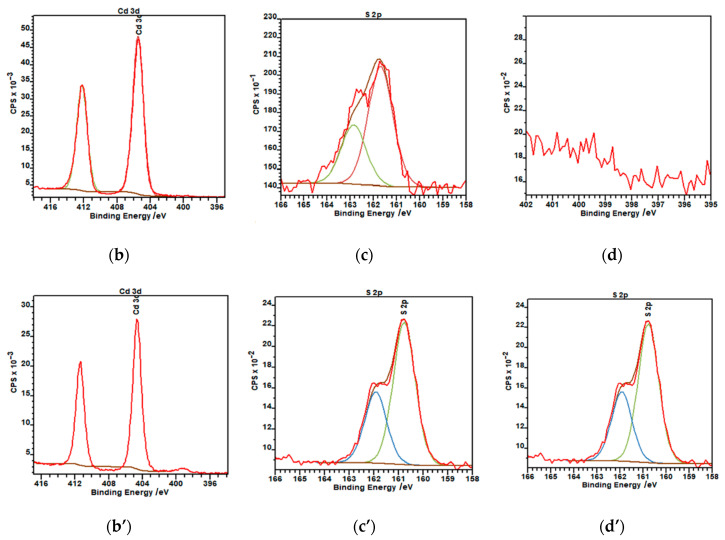
X-ray photoelectron spectroscopy (XPS) spectra of CdS nanoparticles: (**a**,**a’**) survey spectra of hydrophilic and hydrophobic nanoparticles, respectively; (**b**) Cd 3d region; (**c**) S 2p region; (**d**) N 1s region for hydrophilic NPs; (**b’**) Cd 3d region; (**c’**) S 2p region; and (**d’**) N 1s region of oleylamine-coated CdS nanoparticles. Red curve—experimental data, green/blue curve—fitting curves.

X-ray photoelectron spectroscopy (XPS) measurements were performed to confirm the chemical binding states of the CdS nanoparticles (Figure 3). The XPS survey spectrum is shown in Figure 3a,a’ for both hydrophilic and hydrophobic CdS nanoparticles, diagnostic regions are highlighted in green. The noticeable difference, as expected, is the presence of a strong peak at 282 eV corresponding to aliphatic C 1s of oleylamine hydrocarbon chain and N1s at 396 eV due to the amine nitrogen of this molecule forming the organic shell in the case of hydrophobic CdS nanoparticles. These peaks are absent in the case of hydrophilic CdS nanoparticles. Small peak at 282 eV visible also in the XPS survey spectrum of hydrophilic nanoparticles (Figure 3a) is due to the fortuitous atmospheric carbon.

Figure 3b–d, show the XPS s for the Cd 3d, S 2p regions, respectively, for the hydrophilic and Figure 3(b’–d’) for hydrophobic CdS nanoparticles. The peaks with a binding energy of ca. 405 and 411.8 eV can be assigned to the Cd 3d_5/2_ and 3d_3/2_ component of CdS. These data match the binding energy of Cd–S bonds [46]. Figure 3c,c’ shows the XPS spectra of S 2p. The binding energy of S 2p can be deconvoluted into two peaks, the doublets at ca. 161 and 162.5 eV for S 2p_3/2_ and S 2p_1/2_, respectively. These peaks can be attributed to the S^2−^ of bulk S atoms and to the S^2−^ of surface S atoms [46]. The binding energies of these peaks remained unaffected by the presence of oleylamine coating. The atomic percentage ratio of Cd:S was close to unity, as expected from the stoichiometry of CdS.

#### 3.1.3. FT-IR Analysis

**Figure 4:**
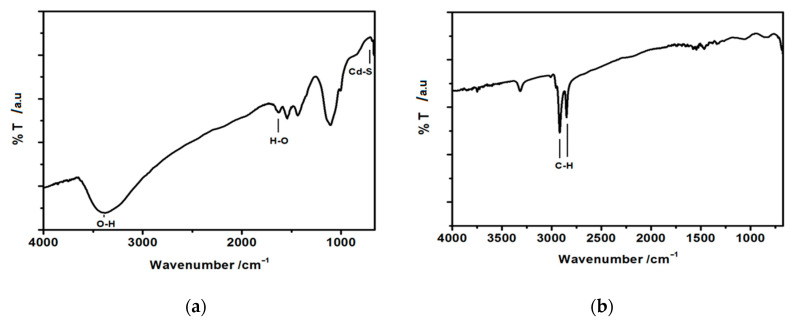
Fourier-transform infrared spectra (FT-IR) spectra of (**a**) hydrophilic, uncoated CdS nanoparticles and (**b**) hydrophobic, oleylamine-capped CdS nanoparticles.

Fourier-transform infrared spectra (FT-IR) spectra were recorded in the range of 675–4000 cm^−1^ for hydrophilic and hydrophobic CdS nanoparticles stabilized with oleylamine. For such measurements, a drop of suspension of uncoated, hydrophilic CdS nanoparticles in water and a drop of suspension of oleylamine-coated CdS nanoparticles in hexane was placed on a gold-covered glass slide and dried. The spectra are presented in Figure 4.

In both cases, the peaks near 700 cm^−1^ arise and can be assigned to Me-S bond [47]. The peak at about 1100–1150 cm^−1^ in the case of hydrophilic nanoparticles (Figure 4a) can be ascribed to the symmetric stretching of C=O bond pointing to some acetate remains in the sample. The peaks observed at 1400 cm^−1^ and 1660 cm^−1^ in the same spectrum (Figure 4a), can be assigned to the vibrational modes of –OH stretching, most probably originating from the adsorbed water molecules from the atmosphere. The broad peak around 3400 cm^−1^ corresponds to the stretching vibration of –OH groups present on the surface of hydrophilic nanoparticles. This peak disappears almost completely in the case of hydrophobic oleylamine-capped CdS nanoparticles (Figure 4b), confirming the formation of oleylamine molecular shell around the CdS core. A weak signal can still be seen at ca. 3350 cm^−1^ in this spectrum. This can be ascribed to the –N-H stretching band of oleylamine. In Figure 4b, the most pronounced peaks, indicative of the organic shell formed around the CdS nanoparticles, are the ones observed at 2940 cm^−1^ and 2850 cm^−1^ and they correspond to –C-H stretching of hydrocarbon chain of oleylamine. Therefore, the FTIR spectra confirm the formation of organic shell around the CdS particles [47].

### 3.2. Morphological Studies of CdS Thin Films

**Figure 5:**
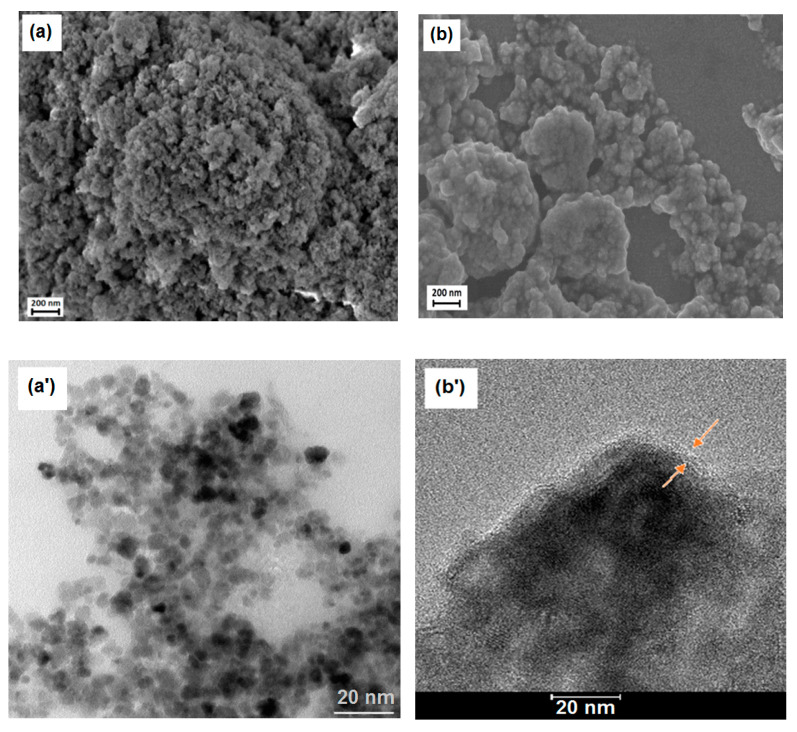

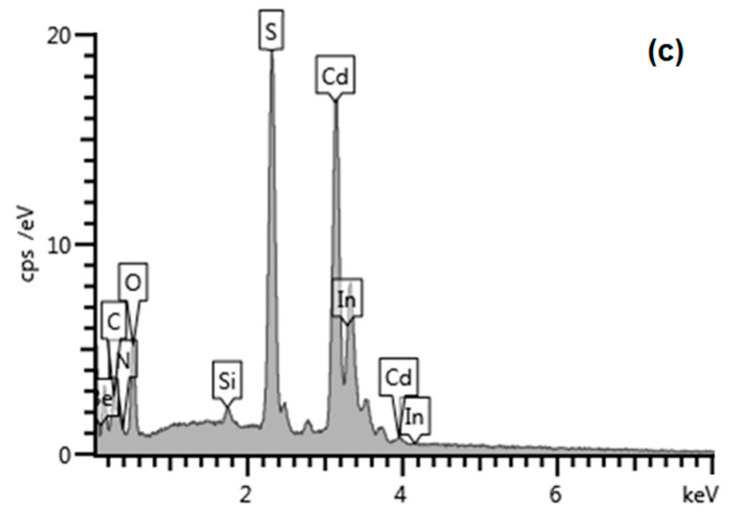
Scanning Electron Microscopy (SEM), (Merlin, Zeiss, Stuttgart, Germany) and Transmission Electron Microscopy (TEM), (Libra 120 Plus, Zeiss, Stuttgart, Germany) images of (**a**,**a’**) hydrophilic CdS nanoparticles (scale bar is 200 nm for SEM and 50 nm for TEM); (**b**,**b’**) hydrophobic CdS nanoparticles deposited onto indium tin oxide (ITO) at 25 mN/m constant surface pressure (L-B transfer, 50 layers, SEM) and TEM of hydrophobic CdS used in the L-B transfer (scale bars: 200 nm SEM and 20 nm TEM); and (**c**) Energy Dispersive X-Ray Spectroscopy (EDS) spectrum of drop-cast CdS nanoparticles on ITO substrate.

**Figure 6:**
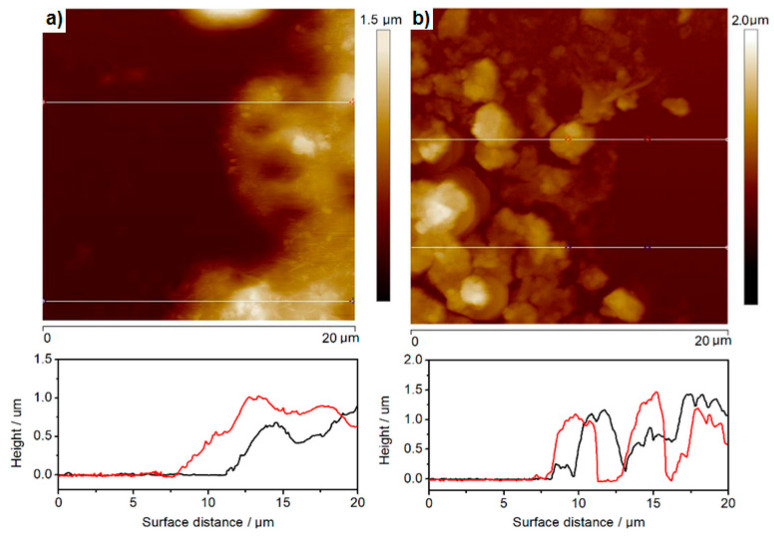
Atomic Force Microscopy (AFM) images and film edge analysis of thickness of (**a**) hydrophobic and (**b**) hydrophilic CdS nanoparticles L-B transferred or drop-cast on ITO, respectively. Red curve—scan forward for the scratching needle, black curve—reverse for the scratching needle.

A representative scanning electron microscopic (SEM) image of hydrophilic CdS nanoparticles (1.67 mg/mL), drop-cast on the ITO surface, is shown in Figure 5a, whereas Figure 5b presents images obtained with Scanning Electron Microscopy for 50 layers of hydrophobic CdS nanoparticles, deposited via the L-B transfer. This image shows aggregates of small nanoparticles, essentially uniform in size. In order to evaluate the average thickness of such aggregates, we subsequently used the AFM, “scratching” (image not shown), as well as the film-edge analysis, Figure 6a,b (the latter technique providing images of better quality), yielding the value within 500 to 800 nm thickness, depending on the measurement point. Therefore, the overall picture that emerges shows generally uniform nanoparticles in tight contact, forming aggregates of different sizes upon drying, with sub-micrometer thicknesses.

As shown also in Figure 5c, the EDS spectrum peaks of CdS on ITO substrate are consistent with Cd, S, O, Si and In as expected for such a sample with some adventitious signals from N and C, most probably from the environmental impurities. The ratio of Cd:S appears to be very close to unity, as expected from the stoichiometry of CdS (atomic % of Cd-30.25 and S-26.11), being congruent with the XPS results. Elemental mapping was also performed (not shown) for Cd and S confirming that the distribution of Cd and S is limited solely to the drop-cast areas visible under SEM.

What can be immediately seen from Figure 5a,b is that in contrary to hydrophilic nanoparticles (Figure 5a), the CdS cores are surrounded by a “cloudy” organic shell that glues the nanoparticles together to form larger aggregates (Figure 5b) with apparently (under this resolution) non-covered ITO surface in between. Zooming in both types of CdS deposits with TEM (Figure 5a’,b’) reveals with more precision the presence of ca. 2–3 nm thick organic layer (red arrows), surrounding individual aggregates of small hydrophobic nanoparticles. Therefore, due to the possible generation of surface states and potential drop across the organic shell molecules adsorbed onto the CdS surface, we expected a substantial hindrance of photopotential and photocurrent, as compared to the hydrophilic, uncoated nanoparticles drop-cast directly on ITO. We will discuss this issue in the next chapter below. We also used the same AFM “scratching” technique (images not shown) and the film-edge analysis, Figure 6a, to assess the average thicknesses of different aggregates. Surprisingly enough, the values obtained for 50 layers deposited via the L-B technique fall within the regime of 500 to 800 nm thickness, depending upon the spot, similar to the uncoated, drop-cast deposits. However, these values have to be considered as a very rough approximation only, due to the soft organic shell surrounding the nanoparticles and interacting with the AFM tip.

Figure 6 presents the AFM images assisted with film-edge analysis of the hydrophobic (a) and hydrophilic (b) CdS deposits that were either L-B transferred or drop-cast on ITO, respectively. The film-edge analysis was done based on two randomly selected spots on each deposit surface as presented by the red and black line thickness profiles in Figure 6. As clearly seen in the cross-sectional analysis of the films, the hydrophobic CdS deposits present more uniform film thickness. This was to be expected since they are being deposited via the L-B technique (50 layers) and glued with oleylamine, while hydrophilic CdS nanoparticles form more porous deposits. The root-mean-square parameter, which is describing the film uniformity, is about 140 nm and ca. 400 nm for L-B and drop-cast CdS films, respectively.

### 3.3. Optical Absorption and Photoluminescence Studies

The optical absorption spectra, which are related to electrons transition from the valence band (VB) to conduction band (CB), were recorded to evaluate the optical bandgap energy, *E_g_*, of the prepared nanoparticles. The Tauc’s equation is presented, where the energy of the optical bandgap *E_g_* of CdS nanoparticles can be evaluated:

A*hν* = K(*hν* − *E_g_*)*^n^*, (2)

where: α is the absorption coefficient of semiconductor, K is the frequency-independent material constant and exponent *n* depends on the nature of transition in the semiconductor, that is, =½ for allowed direct transition [45].

**Figure 7:**
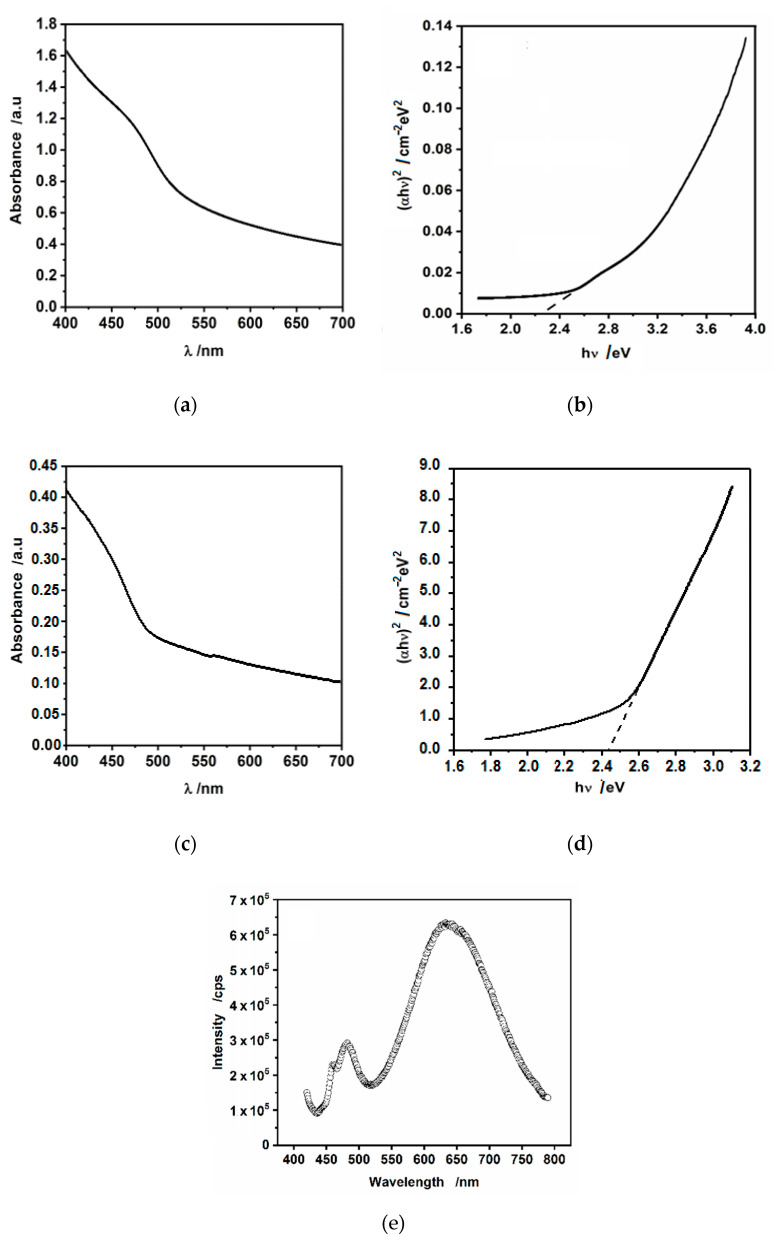
(**a**) UV-vis spectrum and (**b**) (α*hν*)^2^ vs. *hν* dependence obtained forhydrophiic drop-cast CdS nanoparticles suspended in pristine water; (**c**) UV-vis spectrum and (**d**) (α*h*ν)^2^ vs. *hν* dependence obtained for the suspension of hydrophobic CdS nanoparticles in hexane; (**e**) PL spectrum of the suspension of hydrophobic CdS nanoparticles in hexane.

UV-vis absorption spectrum of hydrophilic CdS nanoparticles is shown in Figure 7a. The corresponding Tauc plots (Figure 7b) yielded the energy bandgap of ca. 2.3 eV, being in good agreement with previous reports [35,47]. As it is evident from Figure 7a,c, there is a blue shift (ca. 30 nm) of the absorbance threshold and subsequent hump in the case of hydrophobic, oleylamine-capped CdS nanoparticles as compared to hydrophilic, uncoated ones. This effect may be due to the sequestering of smaller CdS nanoparticles by oleylamine (present only in the hexane phase) from the aqueous phase, leaving larger nanoparticles in water. This results in an increase in the band-gap energy due to an increase in the surface confinement. This is also evident in Tauc plots, Figure 7b,d, where the estimated band-gap energy increased slightly from 2.3 eV to ca. 2.45 eV.

Photoluminescence spectroscopy was used to further investigate the properties of CdS nanoparticles, because it is sensitive to quantum confinement and defect states. The luminescence spectrum of oleylamine-stabilized CdS nanoparticles suspended in hexane is shown in Figure 7e. We were unable to record the photoluminescence (PL) spectrum of hydrophilic nanoparticles, because of the large scattering of incident light and precipitation. Nevertheless, in our previous work [43], we have shown the PL spectrum of hydrophilic CdS nanoparticles drop-cast on quartz substrate. In that work, we have argued that the single peak appearing at ca. 525 nm (excitation 405 nm) corresponds to the near-band edge (green) emission. Here, Figure 7c shows two bands at excitation 405 nm: a narrow one at 481 nm (2.58 eV) and a wide band at 635 nm (1.95 eV). A small band at ca. 470 nm is most probably due to the Raman scattering, because it moves along the wavelength axis with a shift of the excitation wavelength. The band at 481 nm is attributable to the band-edge emission (electron-hole recombination) shifted towards blue by ca. 44 nm (0.22 eV) as compared to the hydrophilic CdS [43]. This shift is again most probably due to surface confinement, however, it may result also from excitonic recombination. For the broad, large peak at 635 nm, we are inclined to assign it to the deep trap defects of surface states. This emission can be also assigned to the presence of many defects, such as sulfur defects or interstitial vacancies [48]. We keep in mind that it is believed that organic ligands passivate the surface trap states but the presence of this peak centered at 635 nm shows that at least some of such surface states are not passivated by oleylamine.

### 3.4. Photoelectrochemical Features of CdS Drop-Cast Hydrophilic Particles and Hydrophobic CdS Particles in L-B Layers

**Figure 8:**
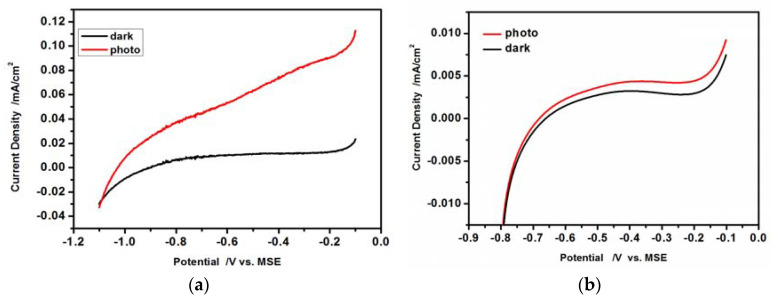
Linear scan curves recorded under dark and illumination vs. Mercurous Sulfate Electrode (MSE) electrode. Scan rate 20 mV/s; (**a**) CdS hydrophilic drop-cast nanoparticles on ITO; (**b**) 50 L-B layers of hydrophobic CdS nanoparticles deposited on ITO.

The photoelectrochemical study was carried out using the hydrophilic drop-cast CdS nanoparticles or L-B layers of hydrophobic CdS nanoparticles deposited on ITO. The photoactivity was measured under visible light illumination in the solution containing redox couple SO_3_^2−^/SO_4_^2−^ (ratio of concentration 2:1). The linear scan voltammetry (LSV), the open circuit potential and chronoamperometric measurements in dark and under illumination were carried out. The resultant LS voltammograms are shown in Figure 8a,b.

The obtained linear scan voltammograms are typical for the n-type semiconductors in the presence of an appropriate redox couple. The dark oxidation currents at *E* > −1.1 V (Figure 8a) and at *E* > −0.70 V (Figure 8b) are very small, because of the very low population of the minority carriers (holes) at the semiconductor/solution interface. Upon illumination of the electrode (Figure 8a,b red curves) the photogenerated electron-hole pairs are separated under the electric field. The holes are subsequently transferred towards the electrode surface and are involved in the oxidation of SO_3_^2−^ to SO_4_^2−^, leading to the increase of the oxidation current. Comparing the results presented in Figure 8a,b, it is clearly seen that the dark current and current under illumination recorded for L-B layers of hydrophobic CdS nanoparticles are much smaller than those in the case of hydrophilic drop-cast nanoparticles.

Moreover, a shift of the potential of the photocurrent onset is observed to more positive values (Figure 8b) in the case of L-B layers of hydrophobic CdS nanoparticles. It is caused by the insulating oleylamine shell around the CdS nanoparticle core producing an additional potential drop at the nanoparticle/electrolyte interface and also increasing the electrode resistance (vide infra).

The shape of current-voltage curves in Figure 8a,b at lower potentials is similar. At potentials lower than −0.9 V (unmodified CdS) and −0.65 V (oleylamine-covered CdS), the increase of cathodic current is observed as a result of hydrogen evolution and participation of majority carriers-electrons in the reaction. The differences in currents values we are inclined to attribute to the resistance of the hydrophobic coating. At potential more positive than −0.9 V (Figure 8a) and −0.65 V (Figure 8b) the dark current is low, as the number of minority carriers (holes) which can take part in reaction of sulfite oxidation is not sufficient. Upon illumination the situation changes as holes are created causing an increase of current. Also in this case the current for the hydrophobic samples is lower as a result of coating resistance. One can also notice a small increase of dark current (Figure 8a,b) at potentials more positive than −0.2 V resulting probably from the oxidation/dissolution of CdS caused by minority carriers holes: CdS + 2h^+^ → Cd^2+^ + S. One may notice also, a small and shallow current decrease around −0.2 V, followed by an increase discussed above. This shallow minimum is observed for oleylamine-covered CdS nanoparticles both in dark and under illumination, therefore it seems to be due to the intrinsic behavior of the system. Since the organic shell is formed by unsaturated long hydrocarbon chains (in terms of macroscale—a dielectric), we are inclined to assign this shallow decrease to the ordering of these chains around this potential value, thus increasing the shell resistance.

It is accepted [49,50,51] that the oxidation of sulfite in alkaline or neutral solution leads to the sulfate as the main product of oxidation reaction (3), however, dithionate can also be formed (4):

(3) $${{\mathrm{SO}}_{3}}^{2-}\;+\;2h^{+}\;+\;2{\mathrm{OH}}^{-}\;\to \;{{\mathrm{SO}}_{4}}^{2-}\;+\;H_{2}O,\;\;\Delta_{r}G^{0}\;=\;-179.09\;\mathrm{kJ}/\mathrm{mol},$$

(4) $$2{{\mathrm{SO}}_{3}}^{2-}\;+\;2h^{+}\;\to \;S_{2}{O_{6}}^{2-},\;\;\Delta_{r}G^{0}\;=\;-7.14\;\mathrm{kJ}/\mathrm{mol},$$

The values of standard Gibbs free energy of these reactions clearly point out that the formation of sulfate is preferable. The reactions are irreversible [50], so during the reduction, the evolution of hydrogen (5) or formation of OH^−^ ions can occur (6):

(5) $$2H_{2}O\;+\;2e\;\to \;H_{2}\;+\;2{\mathrm{OH}}^{-},\;\;\Delta_{r}G^{0}\;=\;159.8\;\mathrm{kJ}/\mathrm{mol}$$

(6) $$2H_{2}O\;+\;O_{2}\;+\;4e\;\to \;4{\mathrm{OH}}^{-},\;\;\Delta_{r}G^{0}\;=\;-154.7\;\mathrm{kJ}/\mathrm{mol}$$

When the electrode is immersed under the open circuit conditions some state of pseudo-equilibrium is attained in dark, characterized by a stationary potential. This stationary potential measured versus the reference electrode is the sum of three potential drops across the interface: the potential drop in the space charge layer of CdS ($\varphi_{SC}$ and the potential drops in the Helmholtz and Gouy-Chapman layers in the electrolyte ($\varphi_{H}$, $\varphi_{G-Ch}$, respectively).

The important parameter determining the photoelectrochemical behavior of CdS in the electrolyte solution is its flat band potential $E_{fb}$ at which $\varphi_{SC}$ is equal to 0. The method often used for determination of the flat band potential involved the measurements of the differential capacity of the electric double layer at semiconductor/electrolyte interface $C_{dl}$ followed by the Mott-Schottky analysis.

This analysis is based on the equation describing the capacitance of the space charge layer $C_{SC}$ in depletion state as a function of $\varphi_{SC}$ and applies the Mott–Schottky plot. The plot presents the relation between the inverse of the square of the measured capacitance of the space charge layer $\frac{1}{C_{SC}^{2}}$ versus (*E* – $E_{fb}$, under the assumption, that applied potential *E* causes the changes only in $\varphi_{SC}$ [$\Delta \varphi_{SC}$ $\gg \Delta \varphi_{H}$,$\Delta \varphi_{G-Ch}$]:

(7) $$\frac{1}{C_{SC}^{2}}\;=\;\frac{2}{e\epsilon \epsilon_{0}N_{D}}\left(E-E_{fb}-\frac{k_{B}T}{e}\right)$$

where: *e* is the electronic charge, ϵ the relative permittivity of SC, $\epsilon_{0}$ the vacuum permittivity, *N_D_* the concentration of donors (n-type *SC*), $k_{B}$ is Boltzman constant and *T* is temperature.

**Figure 9:**
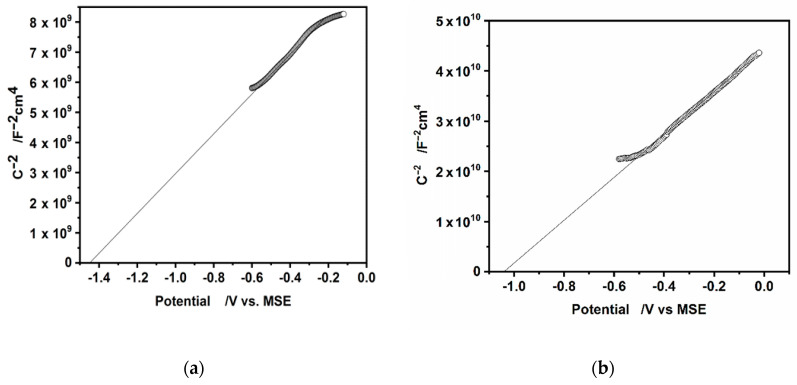
The Mott-Schottky plots for (**a**) hydrophilic and (**b**) hydrophobic, oleylamine-coated CdS nanoparticles. Potentials are quoted vs. MSE electrode.

In practice, the Mott-Schottky plot is usually the graph of $\frac{1}{C_{dl}^{2}}$ as a function of applied potential *E*. The $E_{fb}$ is determined from the intercept of the linear part of the Mott-Schottky plot with the potential axis, $E_{fb}$ = *E* − $\frac{kT}{e}$, ($\frac{kT}{e}$ = 0.026 V, at *T* = 298 K). The Mott-Schottky plots for the hydrophilic and hydrophobic CdS nanoparticles deposited on a glassy carbon electrode are shown in Figure 9a,b.

The values of flat band potentials estimated from these plots are as follows: $E_{fb}$ is equal to −1.44 V, −0.8 V (vs. MSE, NHE respectively) for hydrophilic particles and −1.04 V, −0.40 V (vs. MSE, NHE respectively) for hydrophobic CdS nanoparticles. Literature refers various values of $E_{fb}$ potential of CdS [51]. They depend on: the method used for $E_{fb}$ determination, the crystal structure of CdS (single crystal or polycrystalline samples), the pretreatment of the CdS surface, the composition of electrolyte solution (the presence of SO_3_^2−^ or S^2−^ ions) and pH higher than 12. Our result for hydrophilic CdS nanoparticles is in an agreement with the value of $E_{fb}$ equal to −1.09 V vs. SCE (−1.44 V vs. Mercurous sulfate electrode (MSE) for single crystal CdS in 0.1 M Na_2_SO_3_ solution [52] or with the value of $E_{fb}$ equal to −0.91 V vs. Saturated Calomel Electrode (SCE), (−1.31 V vs. MSE) obtained for polycrystalline CdS, in solution with pH in the range 6–10, [51]. In both cited articles the Mott-Schottky analysis was used.

The shifting of $E_{fb}$ potential to more positive values for hydrophobic CdS nanoparticles is in good agreement with the shifting of the onset potential of photocurrent observed in Figure 8b for L-B hydrophobic nanoparticles. As was mentioned above, this shift is caused by the additional resistance of oleylamine coating. This is also evidenced by the values of resistance determined together with capacitance during the AC voltammetry that differ significantly, being in the range of hundreds ohm and tens of kΩ for hydrophilic and hydrophobic CdS nanoparticles, respectively. The difference between open circuit potential (OCP) in dark (OCP in the range from −0.7 V to −0.75 V vs. MSE) and $E_{fb}$ potential is large. It pointed out, that upon the immersion in the solution of the electrode covered with different kinds of CdS nanoparticles, the inversion state is probably created in the space charge layer of semiconductor.

**Figure 10:**
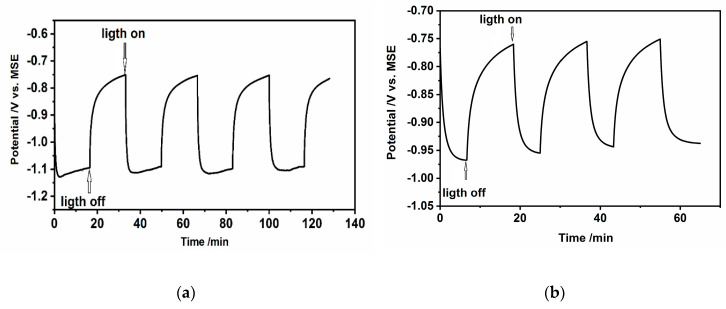
Changes of open circuit potential during illumination of (**a**) CdS hydrophilic drop-cast nanoparticles on ITO, (**b**) L-B layers of hydrophobic CdS nanoparticles deposited on ITO (50 layers) in 0.2 M Na_2_SO_3_/0.1 M Na_2_SO_4_.

In the inversion state, the space charge layer of n-CdS is positively charged due to the charge of minority carriers–holes and the band bending connected with the potential energy of electrons is negative in the potential scale ($\Delta \varphi_{SC}<0)$. During the illumination at the open circuit conditions (OCP), the electron-hole pairs are created and separated in the electric field leading to the changes in the total concentration of electrons and holes (with a relatively much larger increase of the hole concentration as minority charges). This results in the changes of the Fermi energy of charge carriers, followed by a decrease of band bending and changes of OCP in the direction of the flat band potential. This is shown in Figure 10a,b These figures also present the transients of OCP in time, under subsequent on/off illumination. The changes of OCP, under illumination, are about 0.4 V and 0.2 V, for hydrophilic drop-cast nanoparticles and hydrophobic nanoparticles organized in L-B layers, respectively.

**Figure 11:**
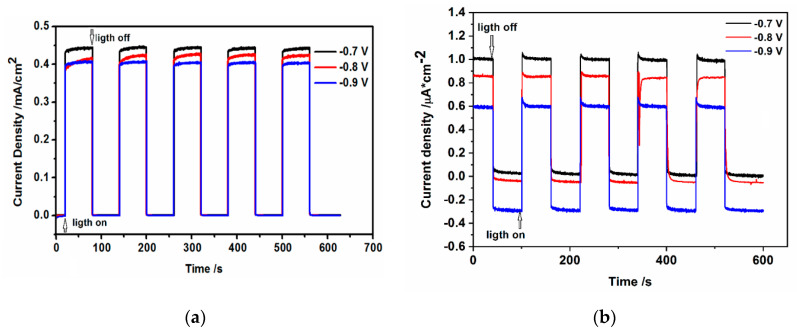
The changes of current densities during illumination at constant potentials of (**a**) CdS hydrophilic drop-cast nanoparticles (**b**) L-B layers (50 layers) of hydrophobic CdS nanoparticles deposited on ITO in 0.2 M Na_2_SO_3_/0.1 M Na_2_SO_4_.

When the CdS hydrophilic drop-cast nanoparticles and the L-B films of oleylamine-coated CdS nanoparticles deposited on ITO (photoanodes) were illuminated under chronoamperometric conditions, the instantaneous, sharp increase of current is observed (Figure 11a,b).

The photocurrent changes, shown in Figure 11a,b depend on the applied potential and slightly increase when the potential becomes more positive. The main differences between these two investigated systems are: (i) very low value of photocurrent, the current is about 450 times lower (at −0.7 V), in the case of hydrophobic nanoparticles organized in L-B layers; (ii) the existence of characteristic “spikes” or “overshoots” on j (current density)–t decay of photocurrent response in the case of CdS-LB layers. The decrease of the photocurrent we ascribe to the organic insulating oleylamine shell of CdS nanoparticles.

### 3.5. Open Circuit Photovoltage and Photocurrent Analysis

The above description, concerning the photogeneration of holes and electrons, followed by their separation in the electric field and further direct charge transfer at photoanode (holes), cathode (electrons) interfaces is very simple and other effects should be considered as well, such as the electron-hole recombination that changes the concentration of charge carriers and can determine their behavior. Two types of recombination can be considered: the inner recombination in nanoparticles (bulk recombination) at dislocation and defects, as well as surface recombination. Dislocations of S^2−^ vacancies or Cd^2+^ ions and defects in CdS nanocrystals form additional energy levels in the bandgap energy of nanoparticles called the traps. The prevailing electrically active defects in the CdS samples are the interstitial Cd atoms providing the n-type conductivity [53]. In the case of surface recombination, the grain boundaries of nanoparticles, the boundaries between CdS nanoparticles and organic shell or ions from the solution adsorbed on the CdS surface can work as traps for charge carriers. The recombination with the participation of traps is a two-step process. In the case of surface recombination, it consists of the capture of an electron from conducting band by surface traps–the empty recombination center and the capture of holes from the valence band. The trapped charge carriers can go further recombination or take part in the redox reaction (charge transfer to the Ox or Red form of redox couple at CdS nanoparticles/ electrolyte interface. Which process occurs depended on charge carrier lifetime and rate constants. Some models were described in the literature by Zaban, Bisquert et al. [54,55], Peter [56] and Peter et al. [57]. These models allowed the determination of the lifetime of electrons as well as the rate constant of charge transfer and recombination. Based on these models and our results (Figure 10a,b φ*_OC_* – f(t) and *J* – f(t) (Figure 11a,b) we discuss the possible mechanisms below.

Photopotential of the electrode can be determined from the differences in the values of OCP in dark and under illumination:

(8) $$E_{ph}\;=\;|E_{oc,l}\;–\;E_{oc,d}|$$

where: $E_{oc,l}$ and $E_{oc,d}$ are the open-circuit potentials (OCP) under illumination and in dark, respectively.

The value of OCP during illumination of n-type semiconductors decreases, going in the direction of flat band potential. Evaluating the results shown in Figure 10a,b, one can distinguish three regions, two of them should be characterized by the constant values of $E_{oc}$ (plateau on the curves $E_{oc}$ vs. t in dark and under illumination) and the third one—by a slow decay of open-circuit voltage when the light is switched off.

**Figure 12:**
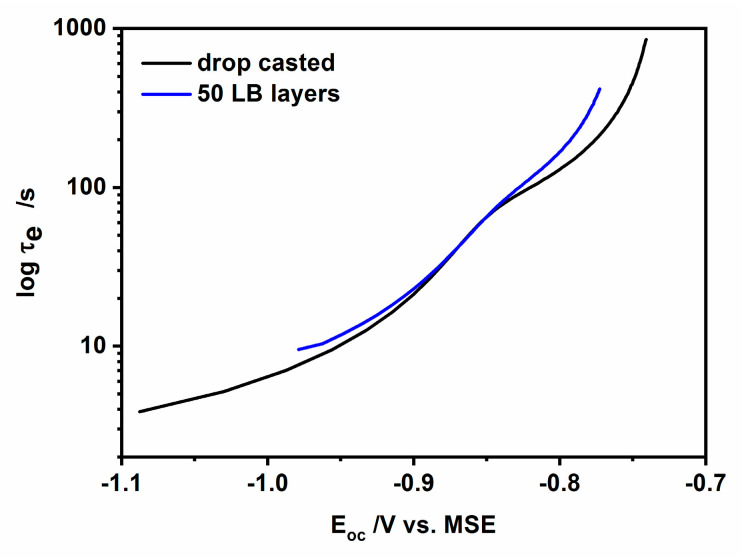
Representation of the electron lifetime in CdS nanoparticles deposited on ITO via drop-cast (hydrophilic nanoparticles) and the L-B transfer (hydrophobic nanoparticles).

Based on the Zaban’s et al. concept [50] the results of $E_{oc}$ decay can be used to estimate the electron lifetime (τ_e_) using the following equation:

(9) $$\tau_{e}\;-\;\frac{k_{B}T}{e}{\left(\frac{dE_{oc}}{dt}\right)}^{-1}$$

where: $k_{B}$ is the Boltzmann constant, *e* is the elementary charge and *T* is the temperature expressed in Kelvins and t is a decay time. This equation allows estimate the overall lifetime of trapped and free electrons upon their photogeneration in semiconductors. The dependence of the electron lifetime, extracted from the experimental results presented in Figure 10a,b as a function of potential is shown in Figure 12. As it is evident from this figure similar profiles were obtained for both types of CdS nanoparticles: hydrophilic and hydrophobic. Two characteristic regions can be distinguished on the curves: the one with low values of $\tau_{e}$ and the other with a high value of electron lifetime. These regions are separated with a hump. We are inclined to assign the hump (at ca. −0.85 V) between these two regions to the transition from the depletion layer, where the bulk trap recombination of carriers is preferable, to the p-type inversion layer with holes as majority carriers, where the dominant role in processes, at a potential close to OCP, play the surface traps.

Comparing the τ_e_ values for both types of nanoparticles (taking into account the logarithmic scale), we can observe that the carrier lifetime is longer in the case of hydrophobic, oleylamine-coated nanoparticles: the closer to the dark open circuit potential values the larger the observed difference. And so, in the case of the potential −0.98 V the difference was only ca. 2 s, while for the potential −0.8 V it reaches 35 s, with slower recombination always for the case of oleylamine-coated nanoparticles. The electron-hole recombination becomes almost identical for drop-cast and L-B transferred CdS nanoparticles at the “hump” at ca. −0.85 V, separating the two different transients that appear at more negative and positive potentials. The observed longer carrier lifetime for oleylamine-coated CdS nanoparticles may be due to the charge trapping at the well-developed interface at the organic shell/CdS nanoparticle and by the resistance of the organic shell against the flux of charges.

Much more information about recombination and competition between recombination and charge transfer process at the CdS/solution interface can be obtained analyzing the photocurrent response to the chopped illumination (Figure 11a,b) at constant potentials (chronoamperometry).

Let us compare the photocurrent results obtained for hydrophilic drop-cast CdS nanoparticles with those for hydrophobic CdS-LB transferred nanoparticles.

In the first case (hydrophilic nanoparticles) one can observe a relatively slow increase of photocurrent before the steady-state is reached. It reflects the final slow step of the build-up of free or surface-trapped holes responsible for further oxidation of the reduced form of redox couple in the electrolyte. When the photocurrent attains its plateau and a steady state is reached, the rate of holes arrivals is exactly balanced by the interfacial charge transfer towards the surface and recombination of carriers. However, the slow accumulation of minority charges at the interface most probably indicates the rapid scavenging of photogenerated minority carriers by the reduced form (SO_3_^2−^) of redox couple from the electrolyte [55]. This assumption is also strongly supported by the absence of any overshoots in the photocurrent response measured during the on/off experiments in the studied potential range, suggesting that the carrier recombination is negligible comparing to the interfacial charge transfer to the redox electrolyte.

**Figure 13:**
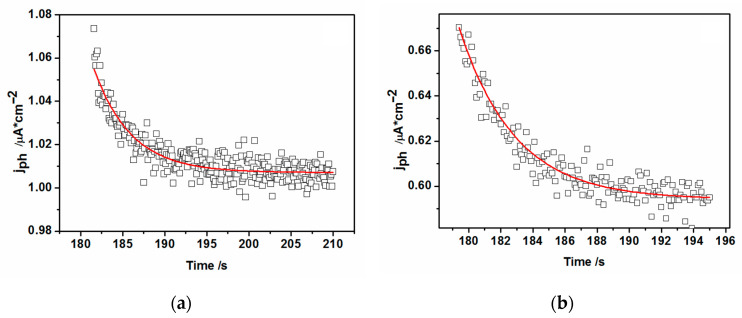
The transients of photocurrent overshoots (spikes) at (**a**) V = −0.7 V and at (**b**) V = −0.9 V (vs. MSE) for the case of 50 Langmuir-Blodgett layers. Experimental data (empty symbols) taken from Figure 8b.

On the contrary to the CdS drop-cast electrodes, in the case of CdS–L-B electrodes after the light “on,” the photocurrent response shows a characteristic “spike” or “overshoot,” followed by a decay to a steady-state condition (Figure 13b). Then, after the light was switched off, the photocurrent dropped back to zero but no overshoot was observed—an untypical behavior since the cathodic current spike should reflect the decay of the surface hole concentration by recombination and interfacial transport [56]. As described in the literature, such behavior can be ascribed to the occurrence of substantial band edge unpinning as a consequence of the accumulation of minority carriers at the interface that changes the potential drop across the Helmholtz double-layer [58]. In our case, we should also consider the influence of the changes in the potential drop within the organic shell. As postulated by L. M. Peter [56], the time-dependent solution of the overshoot can be easily obtained, yielding the values of charge recombination rate constant *k_rec_* and charge transfer rate constant *k_tr_.*

Resolving the “on” transients shown in Figure 13a,b and applying the approach found in the literature [52], one can get the characteristic decay time dependent of both the recombination and charge transfer processes, τ, to be close to 4.4 s for −0.7 V and 3.3 s for −0.9 V applied potentials from the fits to the experimental photocurrent values. These decay times can be used for subsequent retrieval of *k_rec_*, *k_tr_*, rate constant values from the following equation:

(10) $$\tau ={\left(k_{rec}+k_{tr}\right)}^{-1}.$$

Therefore, the sum of *k_tr_ + k_rec_* can be evaluated as 0.23 s^−1^ and 0.3 s^−1^, respectively. According to this model, *k_tr_* and *k_rec_* are the nanoparticle/solution hole transfer and recombination time constants, respectively.

Subsequently, knowing the normalized ratio of *j*_∞_ to *j*_0_:

(11) $$\frac{j\;\left(\infty \right)}{j\left(0\right)}=\;\frac{k_{tr}}{k_{tr}+k_{rec}},$$

where: *j*(∞) and *j*(0) are current densities at steady state (∞) and t = 0, we can conclude for L-B layers of hydrophobic nanoparticles, that the value of hole transfer rate constant *k_tr_* equals 0.22 s^−1^ and *k_rec_* = 0.01 s^−1^ for −0.7 V, whereas *k_tr_* = 0.27 s^−1^ and k_rec_ approximates the value of 0.03 s^−1^ for −0.9 V. The observed increase of recombination rate constant with the potential decrease agrees with a decrease of electron lifetime (see Figure 12), when the potential is changed toward the negative direction. This effect is more pronounced in the case of the oleylamine-coated CdS nanoparticles.

This may result from the decrease of band bending and withdrawal of holes from the space charge region of CdS or CdS/oleylamine interface. In the case of CdS/oleylamine interface this withdrawal is even stronger.

As can be seen from the data retrieved from Figure 13, the slowest process for both potentials is the recombination of the photogenerated electron-hole pairs, whereas the charge transfer kinetics is ca. 10 times faster. Therefore, we are inclined to assign the photocurrent overshoot decay time mostly to the recombination process. Both models illustrated in Figure 12 and Figure 13 point out that the presence of oleylamine shell surrounding the CdS nanoparticle core results in the substantial hindrance of photoactivity in the case of L-B CdS films. This hindrance is most probably due to the resistance and potential drop across the dense organic film of oleylamine, inhibiting the charge transfer of minority charges (holes) to the redox solution and also slowing the electron-hole recombination process apparently by creating more charge carrier traps within the hydrophobic nanoparticles.

Our values of *k_tr_*, which describe the photooxidation of sulfites are about 1000 times lower than those presented in the literature for CiGSe/(thin layer) CdS system in the solution containing Fe(CN)_6_^3−^/Fe(CN)_6_^4−^ redox couple (*k_tr_* = 10^2^ s^−1^) [55,58]. In our case, the charge transfer across the interface is largely blocked by the presence of the organic layer around the CdS nanoparticles. Moreover, the photooxidation of sulfites is probably much more complicated than the one-hole oxidation reaction of Fe(CN)_6_^4−^. The dominant influence of the organic layer is pointed out by the differences in photocurrent recorded for hydrophilic CdS drop-cast and hydrophobic CdS L-B samples (about 500 times difference). Therefore, due to the possible generation of surface states and potential drop across the organic shell molecules adsorbed onto the CdS surface, we observed a substantial decrease of photopotential and photocurrent, as compared to the hydrophilic, uncoated nanoparticles drop-cast directly on ITO, in which case the slow step of the build-up of free or surface-trapped holes responsible for further oxidation of the reduced form of redox couple in the electrolyte is observed.

## 4. Conclusions

Photoelectrochemical behavior of CdS semiconductor nanoparticles of ca. 5.6 ± 1.2 nm diameter stabilized with oleylamine monolayer, was compared with pristine CdS nanoparticles in an attempt to analyze the effect of organic coating on the performance of such a nanostructured semiconductor. Comparative photoelectrochemical studies of both types of nanoparticles on ITO electrodes were performed in an aqueous solution containing SO_4_^2−^/SO_3_^2−^ redox couple. The electron lifetime in both hydrophilic and hydrophobic nanocrystalline CdS was determined, revealing longer carrier lifetime for oleylamine—coated CdS nanoparticles, supposedly due to the charge trapping at the well-developed interface at the organic shell/CdS nanoparticle and due the dominant influence of the resistance of the organic shell against the flux of charges. Substantial hindrance of photopotential and photocurrent was observed for L-B CdS films as compared to the hydrophilic, uncoated nanoparticles drop-cast directly on ITO. The obtained photocurrent and photopotential for CdS L-B electrodes are significantly lower (photopotential about two times and photocurrents about 500 times for the electrode with 50 L-B transfers).

To get more information about the photoelectrochemical activity of oleylamine-coated CdS nanoparticles, we resolved the “on” transients of the photocurrent response. This feature was observed only in the case of oleylamine-covered CdS nanoparticles and can be ascribed to the occurrence of substantial band edge unpinning as a consequence of the accumulation of minority carriers at the interface that changes the potential drop across the organic shell. We obtained the potential-dependent rate constants of the redox processes occurring at the interface, *k_tr_* and charge recombination rate constants, *k_rec_*. The photooxidation of the solution redox couple by holes is significantly less effective than the literature reports on unmodified CdS and results from the blocking behavior of the organic shell. Therefore, the observed decrease of photopotential and photocurrent results from to the possible generation of surface states/traps and potential drop across the oleylamine shell adsorbed onto the CdS surface, as compared to the hydrophilic, uncoated nanoparticles drop-cast directly on ITO. Thus, the long-chain aliphatic molecules not only hinder the charge transfer across the interface but also increase the excited state recombination time. Our studies revealed that the dynamics and efficiencies of excited state electron transfer and recombination processes can be controlled by the presence of suitably chosen organic shell molecules. These observations are of particular importance in light of the development of SSCs, where such molecules can be used to organize, electronically link and protect semiconductor nanostructures on the solar cell electrodes.
